# A one-step method for covalent bond immobilization of biomolecules on silica operated in aqueous solution†
†Electronic supplementary information (ESI) available. See DOI: 10.1039/c8sc02565g


**DOI:** 10.1039/c8sc02565g

**Published:** 2018-08-24

**Authors:** Yong-Kyun Sim, Heetae Jung, Su Hyun Kim, Jung-Woo Park, Woo-Jin Park, Chul-Ho Jun

**Affiliations:** a Department of Chemistry , Yonsei University , 50 Yonsei-ro, Seodaemun-gu , Seoul 03722 , Republic of Korea . Email: junch@yonsei.ac.kr; b Center for Catalytic Hydrocarbon Functionalizations , Institute for Basic Science (IBS) , Daejeon 34141 , Republic of Korea

## Abstract

A simple, one-step method for covalent bond immobilization of biomolecules on silica operated in water is described. In the approach, an NHS-ester linked methallylsilane is utilized as a bifunctional linker to couple the biomolecule to the silica surface. Weak organic acid such as acetic acid activates the silica surface enough to react with bifunctional linker without destroying activity of biomolecule.

## Abstract 2

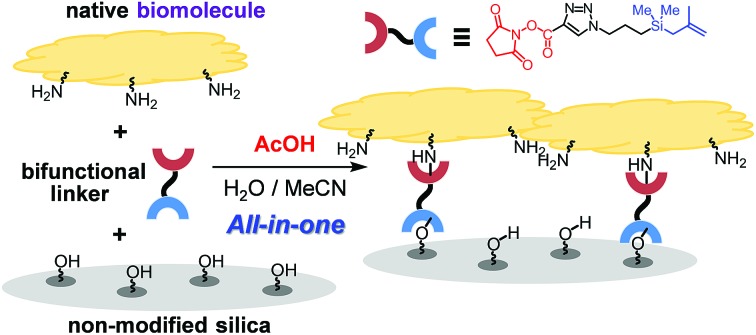
One-step covalent bond immobilization of biomolecules on silica in water is promoted by weak organic acid using bifunctional linker.

## Introduction

**Fig. 1:**
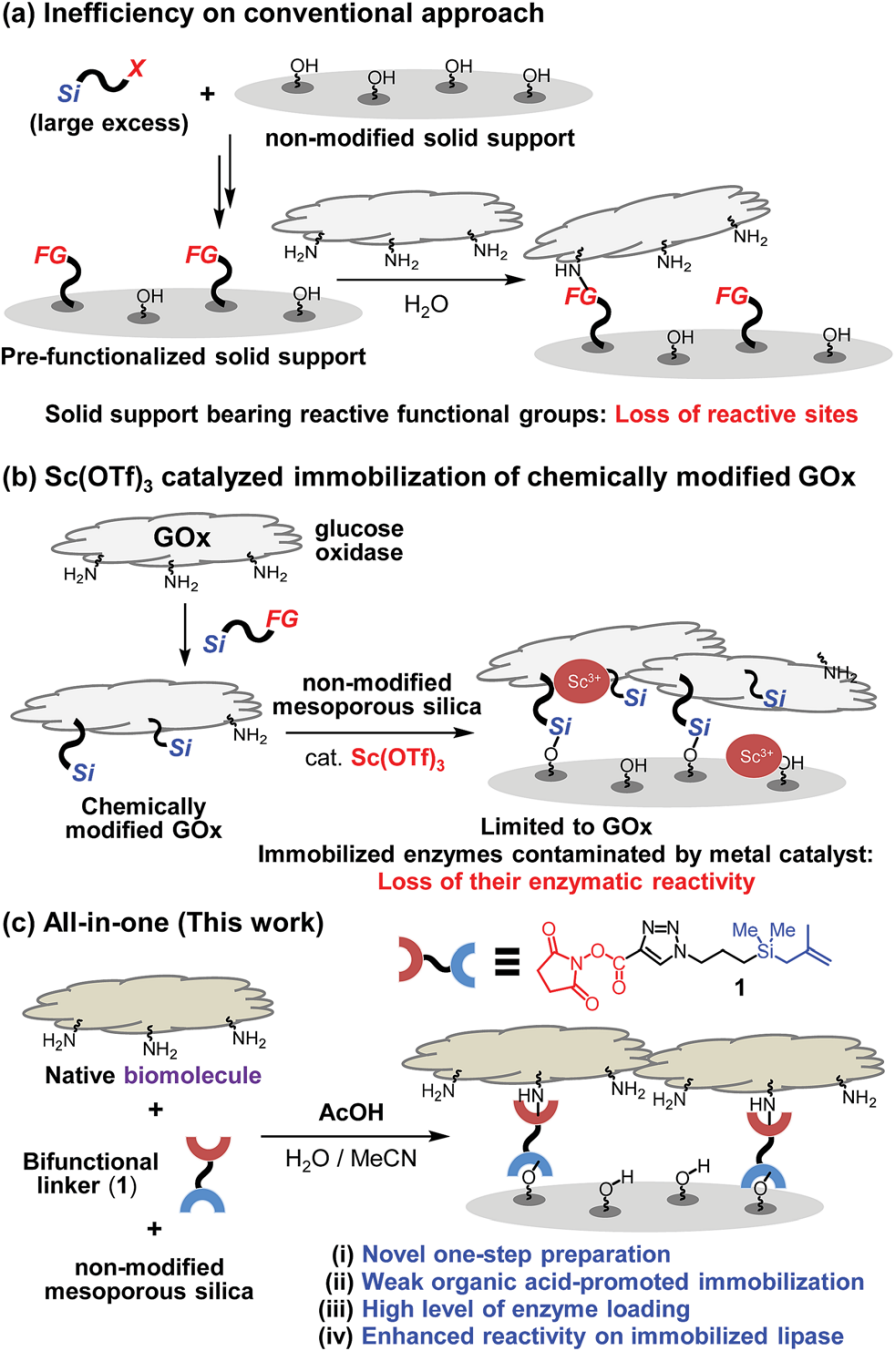
(a) Conventional multi-step methods on biomolecule immobilization. (b) Sc(OTf)_3_-catalyzed two-step immobilization of chemically modified GOx. (c) All-in-one method (this work) on covalent bond immobilization of biomolecules.

While significant advances have been made in fabricating and utilizing immobilized systems, the development of novel techniques for efficient immobilization of biomolecules on inorganic supports requires additional attention.^1^ The establishment of convenient protocols for this purpose would enable ready access to important systems such as chemical reactors and biosensors.^2^ Conventional covalent bond immobilization protocols typically require the use of solid supports that have pre-installed reactive functional groups, including aldehydes, epoxides and NHS-esters, excess amounts of silane precursors, and multi-step processes (Fig. 1a).^3^ Because modified silica supports typically contain much smaller numbers of reactive functional groups in contrast to the numbers of indigenous Si–OH groups present in non-modified silica, direct coupling of biomolecules with non-modified silica in theory should furnish higher degrees of surface coverage. Guided by this expectation, a recent investigation was conducted to explore a two-step method involving immobilization on non-modified mesoporous silica of pre-made, chemically modified glucose oxidase (GOx) promoted by transition metal catalysts such as Sc^3+^ (Fig. 1b).^4^ While this two-step protocol provides an expedient platform to prepare GOx-functionalized silica, the scope of biomolecules using this protocol was determined to be narrow (*vide infra*).

One key point on designing a convenient protocol for efficient biomolecule immobilization on non-modified silica is uncovering a one-step process that operates in aqueous solution. However, there are several difficulties on the development. Commercial silane precursors, such as chloro- or alkoxy-silanes are not suitable for use in this approach due to their poor stabilities in water.^5^ Another issue confronting the development of a direct biomolecule immobilization technique is the need to avoid the use of transition-metal catalysts. This problem was highlighted by the results of our preliminary studies that transition-metal (*e.g.*, Sc^3+^) catalyzed immobilization of biomolecules (*e.g.*, lipase) results in immobilized enzymes displaying diminished activities (Fig. 1b and see below).^6^

To overcome these limitations, we design a protocol, which utilizes weak organic acid promoters like acetic acid, to bring about one-step immobilization of biomolecules on non-modified silica. Below, we describe the results of this effort, which led to the development of a novel approach for one-step, covalent bonding of native biomolecules (proteins and enzymes) to non-modified silica supports, which utilizes a bifunctional, methallylsilane containing linker and does not employ transition metal catalysts (Fig. 1c). Compared to the previously reported two-step protocol, this one-step protocol simplifies the overall process by removing the step involving chemical modification of enzyme. Significantly, owing to its low acidity, acetic acid activates the silica surface for reaction with a bifunctional linker without destroying the activities of biomolecules, leading to broad scope of applicable enzymes.

## Results and discussion

To explore the novel one-step protocol, the designed NHS-ester linked methallylsilane **1** is utilized as a bifunctional reagent. The new method takes advantages of the unique properties of the bifunctional NHS-ester linked methallylsilane **1**, where the NHS-ester group in this reagent reacts with amine residues on biomolecules to create amide bonds and the methallylsilane reacts with Si–OH groups on the silica surface to create Si–O–Si bonds (Fig. 1c). Linker **1** was prepared by Cu-catalyzed [3 + 2] cycloaddition using 2,5-dioxopyrrolidin-1-yl propiolate and 3-azidopropyldimethylmethallylsilane (see ESI† for the detail.). Glucose oxidase (GOx) was chosen as a model protein for the initial phase of the current immobilization studies.

**Fig. 2:**
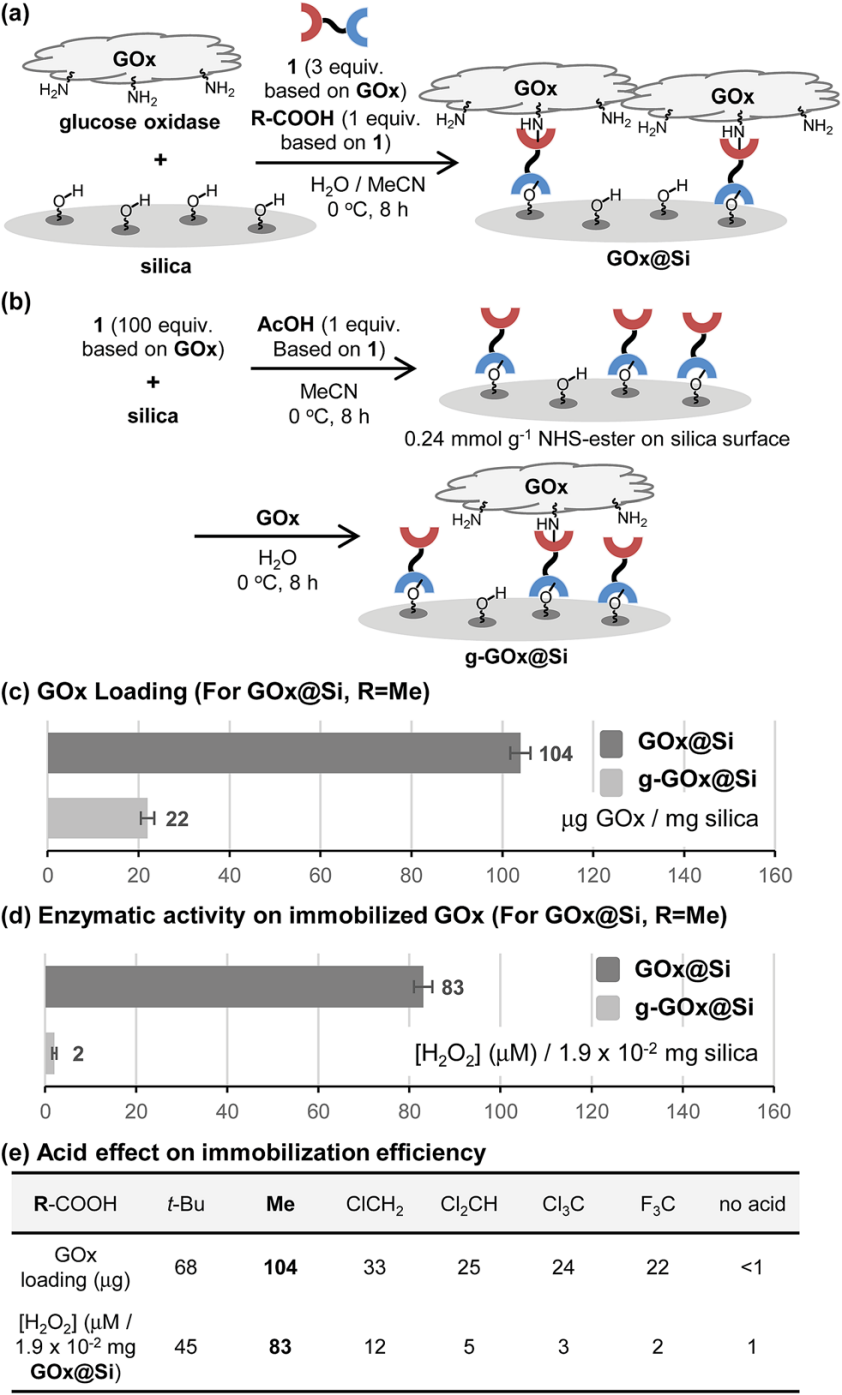
Schematic illustrations for (a) one-step immobilization of GOx to form **GOx@Si** and (b) two-step immobilization to form **g-GOx@Si**. Comparison of (c) GOx loadings and (d) enzymatic activities between **GOx@Si** and **g-GOx@Si**. (e) Acid effect on the one-step immobilization efficiency.

Reactions of glucose oxidase (GOx) and non-modified silica in the presence of the bifunctional linker **1** were carried out with employing acids as catalysts (Fig. 2a). Reaction of **1** with GOx (5 mg) and silica (20 mg, particle size: 110 μm, pore size: 100 nm, area: 26 m^2^ g^–1^) in the presence of acetic acid (1 equiv. based on **1a**) in H_2_O/MeCN at 0 °C for 8 h was found to generate **GOx@Si** with a GOx loading content, determined using a Bradford assay, of 104 μg mg^–1^ silica (Fig. 2c). The enzymatic activity of **GOx@Si** was determined to be 83 μM by utilizing spectroscopic assay for H_2_O_2_ generation *via* reaction of **GOx@Si** (1.9 × 10^–2^ mg), O_2_ and glucose (1 mM) (Fig. 2d).

A stepwise method (grafting method) was employed to carry out GOx immobilization (Fig. 2b) for comparison purposes. In this approach, NHS-ester-functionalized silica, **NHS@Si** (0.24 mmol g^–1^ loading), was first prepared by reaction of **1** with silica in the presence of acetic acid (1 equiv. based on **1**)^7^ and the product was reacted with GOx to generate **g-GOx@Si**. The GOx loading in this material is 22 μg mg^–1^ silica, a value that is 4.7 times lower than that of **GOx@Si** produced using the one-step process (Fig. 2c). It is important to note that the one-step immobilization method for preparation of **GOx@Si** requires a much smaller quantity of bifunctional linker **1** (1.1 mg for 20 mg silica) than is needed for the grafting method (36.4 mg for 20 mg silica).

The results of additional studies showed that the choice of organic acid is crucial for the success of the immobilization process. Specifically, acetic acid displays a superior efficiency, while pivalic acid (*t*-BuCO_2_H) has a lower immobilization efficiency (Fig. 2e). Moreover, strong organic acids such as chloro-, dichloro-, trichloro- and trifluoro-acetic acid are much less effective promoters of this process than is acetic acid.

**Fig. 3:**
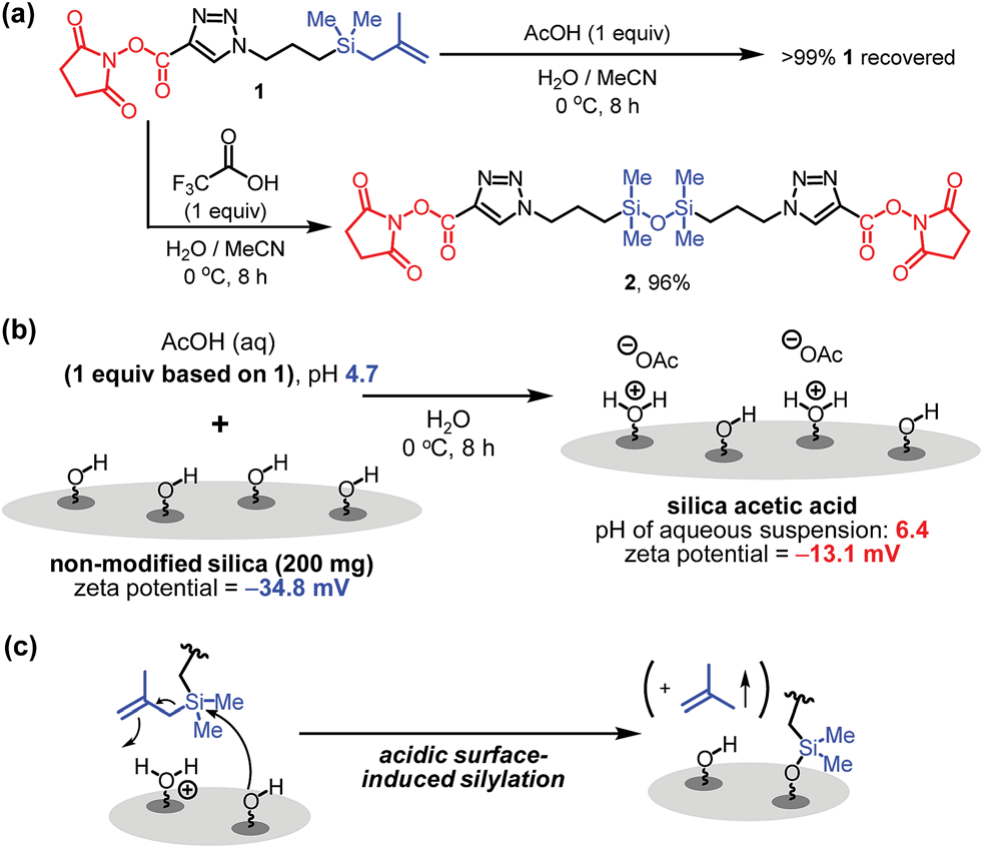
(a) Effect of organic acids on bifunctional linker **1**. (b) pH and zeta-potential of silica acetic acid. (c) Proposed mode of activation of methallylsilanes.

Although it is common for methallylsilane to be activated for reactions by using Lewis acidic metal catalysts or strong Brønsted acids,^8^ how weak organic acids promote immobilization reaction of **1** on silica is intriguing. To gain information about this issue, the influence of acid on the stability of the bifunctional linker **1** in water was assessed. When present in a solution of acetic acid in H_2_O/MeCN (3/1) at 0 °C for 8 h, **1** for the most part remains unreactive. However, bifunctional linker **1** undergoes dimerization to form siloxane **2** in 96% yield when treated with highly acidic trifluoroacetic acid in aqueous acetonitrile (Fig. 3a). Thus, in the presence of trifluoroacetic acid the solution has a significantly high acidity to promote formation of silanol by protodesilylation of **1** with water serving as a silophile. Also, the results show that the presence of silica is required for **1** to undergo reaction in aqueous acetic acid solutions.

To further understand the nature of the acetic acid catalytic process, the pH and zeta-potential of acid treated silica suspensions were determined. The results show that addition of silica (200 mg) to an aqueous acetic acid solution (0.3 mg acetic acid/mL H_2_O, 6 mL) leads to an increase in pH from 4.7 to 6.4. Also, addition of acetic acid to aqueous silica suspension induces an increase in the zeta-potential from –34.8 to –13.1 mV (Fig. 3b). These observations suggest that protonation of the silica surface occurs in acidic solutions to form oxonium ions (*i.e.*, protonated silanols), a process that causes a decrease in the concentration of hydronium ion in the solution and an increase in the population of protonated silanols on the silica surface.^9^ Thus, on the basis of our observations (Fig. 2b, 3a and b), we believe that the protonated silanol groups on the silica surface serve as general acid catalysts and the neutral silanol groups participate as silophiles in simultaneous Si–C bonding cleaving and Si–O bond forming reactions of **1** with silica (Fig. 3c).

**Fig. 4:**
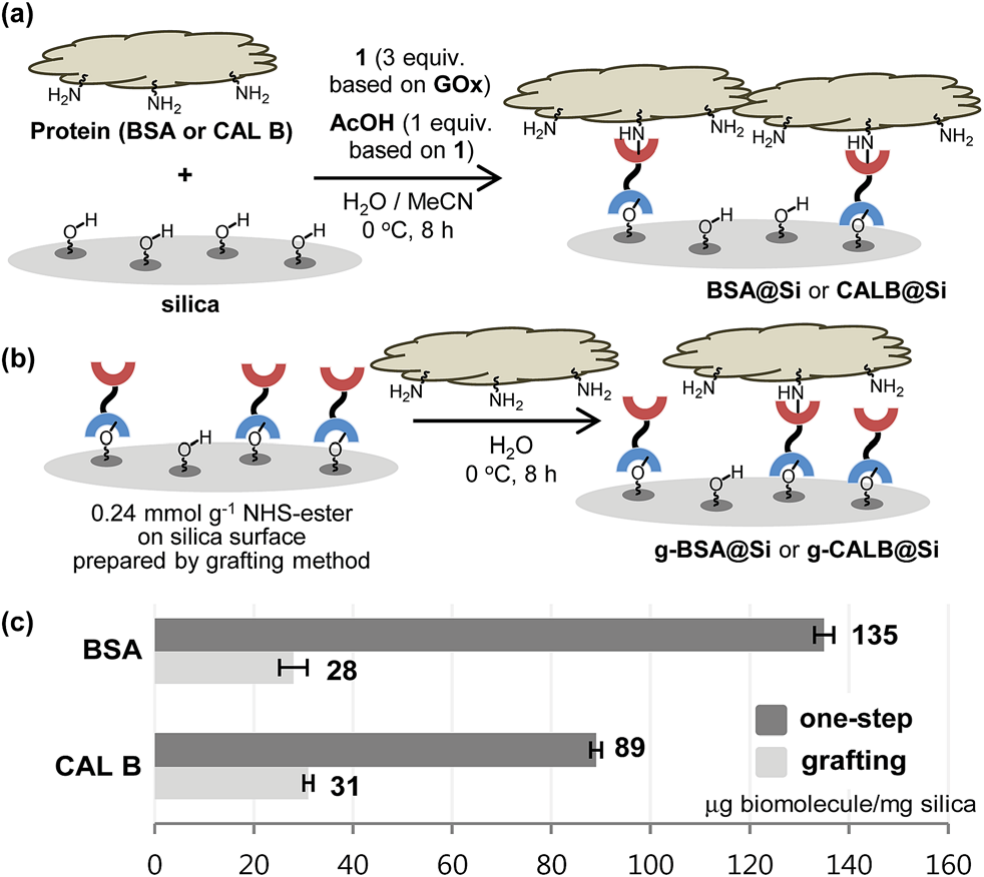
Application to one-step immobilization of BSA and CAL B onto silica.

With the results described above serving as a guide, immobilization reactions of other biomolecules (protein or enzyme) on a silica support were investigated using the optimized conditions (Fig. 4). Immobilization of bovine serum albumin (BSA), which is used in diverse sensor and catalysis applications,^10^ on silica using both the one-step and the grafting method were carried out. As expected, **BSA@Si**, generated using the one-step method has a much higher degree of loading (135 μg) than does **g-BSA@Si** (28 μg).

**Fig. 5:**
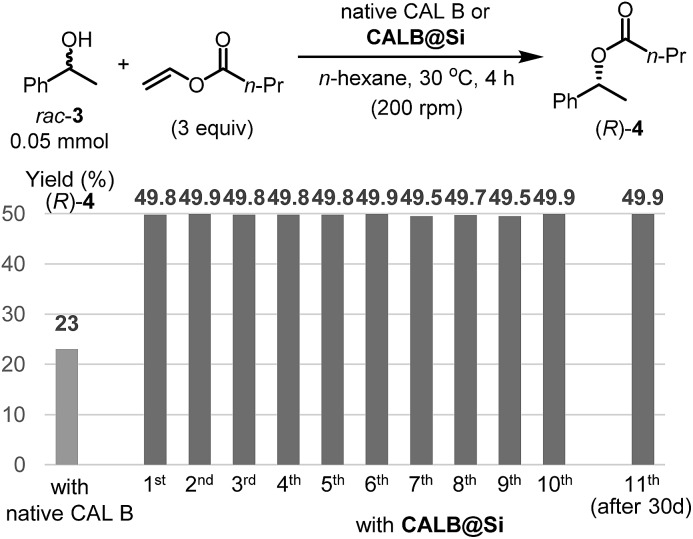
Application to kinetic resolution transesterification reaction using native CAL B and **CALB@Si** and the recyclability of **CALB@Si**. Enantiomeric excesses of the (*R*)-**4** using both native CAL B and **CALB@Si** revealed to be >99% in all experiments.

Another enzyme explored in this effort is *Candida antarctica* lipase B (CAL B), which is known to display excellent levels of enantioselectivity in kinetic resolution (KR) promoting transesterification processes.^11^ The results show that again the CAL B immobilized by using the one-step method (**CALB@Si**) has a much higher degree of loading (89 μg) than does **g-CALB@Si** (31 μg), generated by utilizing the two-step grafting method. The resulting **CALB@Si** along with native CAL B were used to promote kinetic resolution guided transesterification reactions (30 °C, 4 h) of vinyl butyrate with *rac*-α-phenylethanol (*rac*-**2**) (Fig. 5). The theoretical maximum conversion of enantioenriched product in this kinetic resolution reaction is 50%. The process using native CAL B (1.78 mg) generates (*R*)-**4** in 23% yield while the one using 20 mg of **CALB@Si**, which corresponds to the same amount (1.78 mg) of native CAL B employed, results in formation of (*R*)-**4** nearly quantitatively with high enantioselectivity (49.8%, >99% ee). Although the exact reason(s) is not clear, a consideration of earlier proposals^12^ suggests that the enhanced reactivity of immobilized **CALB@Si** is a consequence of incorporating the enzyme into a confined structure. Moreover, **CALB@Si** can be reused to catalyze the KR process 10 times without loss of activity (>99% ee, *E* > 200). Finally, storing **CALB@Si** for 30 days at room temperature does not cause a loss of its KR activity. A notable observation made in this investigation is that one-step immobilization of CAL B on silica, promoted using Sc(OTf)_3_, leads to an immobilized enzyme that has nearly the same loading (76 μg mg^–1^ silica) as that produced using the aqueous acetic acid catalytic system but has a significantly lower ability to promote KR reaction of vinyl butyrate with *rac*-**2** (12.2% (*R*)-**4**, 63.8% ee). The lower KR promoting ability of **CALB@Si** using Sc(OTf)_3_ as the catalyst might be a consequence of detrimental influence of the residual scandium (Sc^3+^) on active sites of the immobilized CAL B.^6^

## Conclusions

The study described above has led to the development of a simple, one-step method for immobilization of biomolecules on unmodified silica. The all-in-one process, which utilizes the bifunctional linker **1** and aqueous acetic acid as the promoter, generates immobilized biomolecules that have high loading capacities and catalytic activities. Importantly, the new protocol can be conveniently applied to immobilization of various biomolecules on silica in water. Especially interesting is the observation that immobilization of CAL B on silica cause an increase in the stability of the enzyme. While further applications to universal biomolecule immobilization are warranted, the new protocol represents a unique strategy for preparation of biomolecule-bound silica using native enzyme and silica.

## Conflicts of interest

There are no conflicts to declare.

## Supplementary Material

Supplementary information
