# Supplementary material for: A one-step method for covalent bond immobilization of biomolecules on silica operated in aqueous solution
Source: Chem Sci. 2018 Aug 24;9(41):7981–5. doi: 10.1039/c8sc02565g (PMC6201817; doi:10.1039/c8sc02565g)
Supplement: Supplementary file 1 [file SC-009-C8SC02565G-s001.pdf]

Electronic Supplementary Material (ESI) for Chemical Science.
This journal is © The Royal Society of Chemistry 2018

Electronic Supplementary Information

A One-Step Method for Covalent Bond Immobilization of Biomolecules on Silica Operated in Aqueous Solution

Yong-Kyun Sim[a], Hee Tae Jung[a], Su Hyun Kim[a], Jung-Woo Park[b], Woo-Jin Park[a], and Chul-Ho Jun[a,*]

[a] Department of Chemistry, Yonsei University, 50 Yonsei-ro, Seodaemun-gu, Seoul 03722, Korea (Republic of)

[b] Center for Catalytic Hydrocarbon Functionalizations, Institute for Basic Science (IBS), Daejeon 34141, Republic of Korea

Contents

I. Materials **S2**

II. Measurements **S3**

III. Experimental **S5**

I. Materials

Glucose Oxidase (GOx, from Aspergillus niger, 147,000 U/g, Mw 80 kDa), Bovine Serum Albumin (BSA, protease free, MW 66 kDa) and Candida Antarctica Lipase B (CAL B, recombinant from aspergillus oryzae, MW 33 kDa) were purchased from Sigma Aldrich. NHS-ester-linked methallylsilane **1** (2,5-dioxopyrrolidin-1-yl 1-(3-(dimethyl(2-methylallyl)silyl)propyl)-1H-1,2,3-triazole-4-carboxylate) was prepared by using the reported procedure (See below for the detailed procedure and characterization data).[1] Acetic acid, chloroacetic acid, trifluoroacetic acid, and pivalic acid were purchased from Sigma Aldrich. Dichloroacetic acid and trichloroacetic acid were purchased from TCI Chemical Company. Silica (particle size: 110 μm, pore size: 100 nm, specific surface area: 26 m^2/g) employed in the immobilization experiments was purchased from Fuji Silysia. Protein colorimetric assay dye was purchased from BIO-RAD. Acetonitrile was distilled by reported procedure prior to use.[2]

Preparation of 1: To an ethyl acetate (270 mL) solution of propiolic acid (2.7 mL (43.4 mmol)) and N-hydroxysuccinimide (5.0 g (34.8 mmol)) was added dropwise N,N'-dicyclohexylcarbodiimide (9.0 g (43.6 mmol)) dissolved in ethyl acetate (30 mL). The reaction mixture was stirred under nitrogen for 8 hours at 0 °C. The mixture was washed with water and brine, dried over anhydrous $MgSO_4$, filtered and concentrated under reduced pressure. The unpurified product (2,5-dioxopyrrolidin-1-yl propiolate) was used in the next step without further purification. To a solution of crude 2,5-dioxopyrrolidin-1-yl propiolate in THF, were added a catalyst mixture of $CuSO_4•5H_2O$ (0.442 g (1.77 mmol)) and sodium ascorbate (0.701 g (3.54 mmol)), and 3-azidopropyldimethy lmethallylsilane (3.5 g (17.7 mmol)), and the mixture was stirred for 4 hours at room temperature. The reaction was quenched with saturated aqueous NH_4Cl, and the mixture was extracted with EtOAc for 3 times. The collected organic layer was dried over anhydrous $MgSO_4$, filtered through Celite pad, then purified by column chromatography (*n*-hexane:ethyl

[1] U.-Y. Jung, J.-W. Park, E.-H. Han, S.-G. Kang, S. Lee and C.-H. Jun, *Chem. Asian J.* 2011, **6**, 638.
[2] *Purification of Laboratory Chemicals*, 4th edition (Eds: W. L. F. Armarego, D. D. Perrin), Butterworth-Heinemann, Oxford, 1996, pp. 77.

acetate=1:1; R_f 0.3) to give 1 (3.8 g, yield: 59 %). Characterization Data for 1: ^{1}H NMR(250 MHz, $CDCl_3$) δ 8.26(s, 1H), 4.53(d, J= 37.5 Hz, 2H), 4.44(t, J= 7.38 Hz, 2H), 2.92(s, 4H), 2.02-1.92(m, 2H), 1.70(s, 3H), 1.63(s, 2H), 0.57-0.50(m, 2H), 0.08(s, 3H); ^{13}C NMR(62.9 MHz, $CDCl_3$) δ 169.0, 156.0, 143.2, 134.9, 129.1, 109.0, 54.1, 27.0, 25.8, 25.3, 12.5, -2.9.

II. Measurements

a. Determination of GOx Activity: Glucose Oxidase (GOx) activity was determined using a GOx assay kit (from Stressgen Biotechnologies) according to instructions provided by manufacturer. GOx-immobilized silica (1.25 mg) was dispersed in Phosphate Buffer Saline (PBS, 1 mL), and the resulting solution was serially diluted. Then, aqueous *D*-glucose solution (1 μM) was added to a diluted solution. After the sample was incubated at room temperature in the dark for 30 min, the supernatant was used for determining the concentration of H_2O_2 generated. A standard solution prepared from 30% H_2O_2 (Calbiochem). Then, the enzyme activity was determined by using the standard curve.

b. Determination of Enzymes Loading Rate: Quantitative measurement of the amount of enzyme immobilized onto silica was carried out by using the Bradford method (Bio-Rad).[3] In this experiment, reactions of NHS-ester-functionalized methallylsilane **1** in acetonitrile (250 μL), enzyme in H_2O (600 μL), silica (20 mg) and acetic acid (0.171 μL) were carried out at 0 ^oC for 8 h, and the resulting protein or enzyme immobilized silica was separated by centrifugation and washed with PBS buffers pH 7 and pH 10 solution (prepared by mixing pH 7 PBS buffer + Na_2CO_3 aqueous solution). The amount of enzyme

[3] Bradford method (Bio-Rad) was used for determining the amount of immobilized enzyme according to the manufacturer's instructions. M. M. Bradford, *Anal. Biochem.* 1976, **72**, 248.

in the washed buffer was analyzed by using the Bradford method. BSA (Bio-Rad) was used as a standard protein.

c. Determination of Lipase Activity: For evaluation of the Candida Antarctica Lipase B (CAL B) activity, the transesterification of vinyl butyrate with *rac*-1-phenylethanol was used. The procedure was performed using the method described in the literature.[4] To a mixture of *rac*-1-phenylethanol (98%, 6.15 μL, 0.05 mmol) and vinyl butyrate (98%, 19.41 μL, 0.15 mmol) in 1 mL of hexane was added lipase-immobilized silica (20 mg). The mixture was shaken at 200 rpm and 30 ^{o}C for 4 h and then analyzed by using a gas chromatography (Agilent 7890A) equipped with chiral capillary column (Rt®bDEXcst, 30 m x 0.25 mm ID, RESTEK).

In the recycling experiments, lipase-immobilized silica was washed with hexane and separated by using centrifugation. An aliquot of the supernatant was assayed. The lipase-immobilized silica was reused and the additional amounts of vinyl butyrate and rac-1-phenylethanol in hexane were added for the 2nd run. The same reaction with the 1st run was performed, and this procedure was repeated 10 times. Following the last run, the lipase-immobilized silica was kept at room temperature for 30 days and then re-assayed.

d. Zeta-potential: An electrophoresis apparatus (Malven Zen 3600 Zetasizer) was used to determine the zeta potential of silica in aqueous suspension. The measurement was repeated three times and the average from them was reported as the final result.[5]

III. Experimental

[4] Y. K. Sim, S. Jung, J. Y. Lim, J. Kim, S.-H. Kim, B. K. Song, B. T. Kim, H. Lee, S. Park, *Tetrahedron Letters.* 2011*,* **52***,* 1041

[5] Guanli, X. et al. *Powder Technol.* 2003, **134**, 128.

a. General procedure for preparation of enzyme(or protein)@Si. A mixture of silica (20.0 mg), NHS-ester-functionalized methallylsilane **1** (3 equiv. based on the lysine residue of enzyme) in acetonitrile (250 μL), acetic acid (1 equiv. based on **1**) and each target enzyme or protein (6.25 x 10^{-5} mmol; 5.0 mg GOx, 4.1 mg BSA, 2.0 mg CAL B) dissolved in 600 μL H_2O was placed into a 1.5 mL Eppendorf tube, and stirred at 0 ^oC for 8 h. The resulting slurry was filtered and washed thoroughly with pH 10 solution (prepared by mixing pH 7 PBS buffer + Na_2CO_3 aqueous solution) and pH 7 PBS buffer solution using centrifugation to give **enzyme(or protein)@Si**.

Note: we performed the control reactions of glucose oxidase (GOx), silica (20 mg) and acetic acid (1 equiv) in the absence of bifunctional linker **1**. After the reaction, the resulting silica was washed with water (1 mL) three times. GOx activity of the silica (0.625 mg, 32x scale described in the manuscript) was measured to be 60.2 μM [H_2O_2], implying some non-specific binding of GOx on silica. However, when the resulting silica was washed three times with pH 10 solution (prepared by mixing pH 7 buffer + Na_2CO_3 aqueous solution) and pH 10 (glycine buffer) buffer solution, any GOx catalytic activities (<5 μM [H_2O_2]/ 0.625 mg sample) was not determined. These results indicate that washing procedure using pH 10 solution removes GOx completely. On the basis of our experimental data, we believe that our experimental protocol including washing procedure on immobilization onto silica avoids non-specific binding of protein.

b. Procedure for preparation of g-enzyme@Si. A mixture of silica (20.0 mg), acetic acid (5.72 μL, 0.10 mmol), **1** (36.4 mg, 0.10 mmol) and acetonitrile (1 mL) was stirred at room temperature for 8 h and then filtered. The precipitate was washed thoroughly with dichloromethane (DCM), methanol (MeOH), deionized water and acetone. The resulting silica was dried under reduced pressure to give **NHS@Si**. The loading efficiency of NHS-ester group in **NHS@Si** was determined to be 0.275 mmol/g NHS-ester groups, based on the N value of elemental analysis (Elemental analysis of **NHS@Si**: C, 3.38; N, 1.54). A

mixture of each enzyme (6.25 x 10^{-5} mmol; 5.0 mg GOx, 4.1 mg BSA, 2.0 mg CAL B) in 600 μL H_2O and **NHS@Si** (20.0 mg) was stirred at 0 °C for 8 h, filtered and washed with with pH 10 solution and pH 7 PBS buffer solution to give **g-enzyme@Si**.

c. Optimization on preparation of GOx@Si.

(a) Several **GOx@Si**s were prepared by reactions of GOx (5.0 mg) and silica (20 mg) with various amount of **1** (**X**= 0.0, 0.5, 1.0, 2.0, 3.0, 4.0, 5.0; based on lysine residues in GOx) in the presence of acetic acid (1 equiv. based on **1**) for 8 h, and evaluated activities of the resulting **GOx@Si**s. As shown in Figure Sx, use of 3.0 equiv. of bifunctional linker **1** was determined to be optimal.

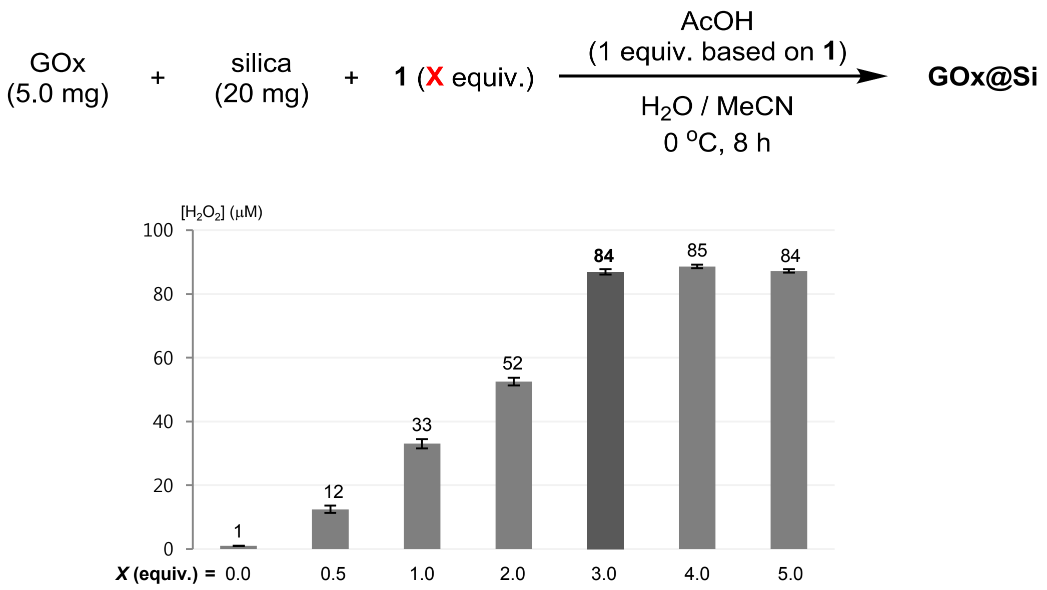

Figure S1. Effect of the amount of bifunctional linker **1** on immobilization efficiency

(b) We next tested the reaction of GOx (5 mg), silica (20 mg) and **1** (3 equiv. based on lysine residues on GOx) with varying the amount of acetic acid (**Y**= 0, 0.1, 0.5, 1.0, 2.0 equiv. based on **1**) for 8 ours, and those activities were measured. As shown in Figure Sx, the use of 1.0 equiv of acetic acid was optimal for this protocol.

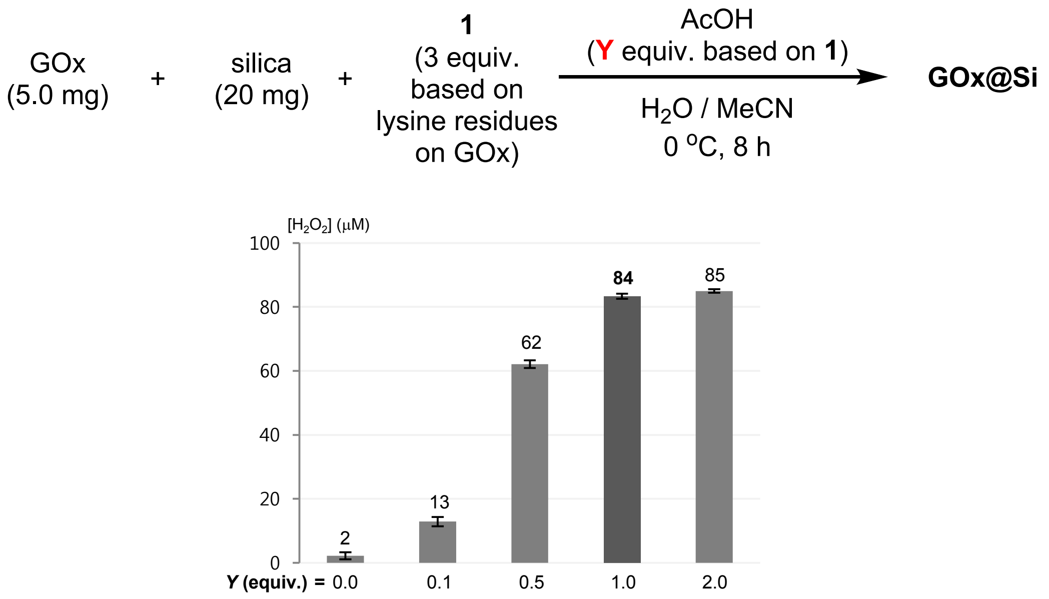

Figure S2. Effect of the amount of acetic acid on immobilization efficiency

(c) Reaction progress was monitored by measuring the activities of **GOx@Si**s prepared from the reactions of GOx (5 mg), silica (20 mg) and **1** (3 equiv. based on lysine residues on GOx) in the presence of acetic acid (1 equiv. based on **1**) for various reaction times (0.5, 1, 2, 4, 6, 8, 10 and 12 h). As shown in Figure S3, 8 h reaction time was determined to be optimal.

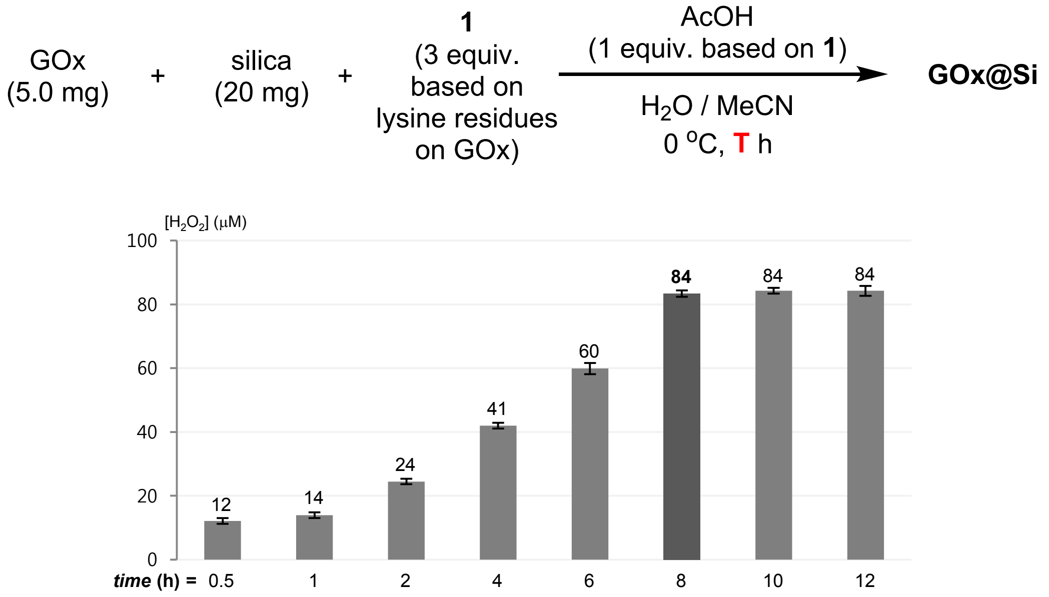

Figure S3. Progress of immobilization reaction

d. Effect of organic acids on bifunctional linker 1 (Figure 3a). A mixture of **1** (2.20 mg, 6 x 10^{-3} mmol), trifluoroacetic acid (0.46 µL, 6 x 10^{-3} mmol) in acetonitrile (250 µL) and H_2O (600 µL) was stirred at 0 °C for 8 h. Extraction with 5 mL of CH_2Cl_2 gave an extract that was dried *in vacuo* to give a residue that was subjected to column chromatography to give siloxane **2** as a colorless liquid in 96 % isolated yield (1.83 mg). **Characterization data for bis(2,5-dioxopyrrolidin-1-yl) 1,1'-((1,1,3,3-tetramethyldisiloxane-1,3-diyl)bis-(propane-3,1-diyl))bis(1H-1,2,3-triazole-4-carboxylate) (2): ^{1}H NMR ($CDCl_3$)** δ =8.37 (s, 2H), 4.42-4.46 (t, J = 7.0 Hz, 4H), 2.90 (s, 8H), 1.91-1.98 (m, 4H), 0.44-0.49 (m, 4H), 0.06 (s, 12H); **^{13}C NMR ($CDCl_3$)** δ = 0.37, 15.06, 24.66, 25.83, 53.76, 129.66, 134.62, 156.01, 169.35; **IR (neat)**: 3519, 2884, 1538, 1434, 1255, 1077, 1048, 840, 702, 652; **HR-MS(ESI+)** calcd. for $[M+H]^+$ $C_{24}H_{35}N_8O_9Si_2{}^+$ 635.2060; found 635.2062.

e. pH and zeta-potential of silica acetic acid (Figure 3b).

The pH of a solution of 1.71 μL (0.03 mmol) of acetic acid in 6.0 mL of water was determined to be 4.50. To this acidic aqueous solution, 50 mg of silica was added repeatedly as shown in Figure S4. The addition of silica resulted in an increase of pH up to 6.45.

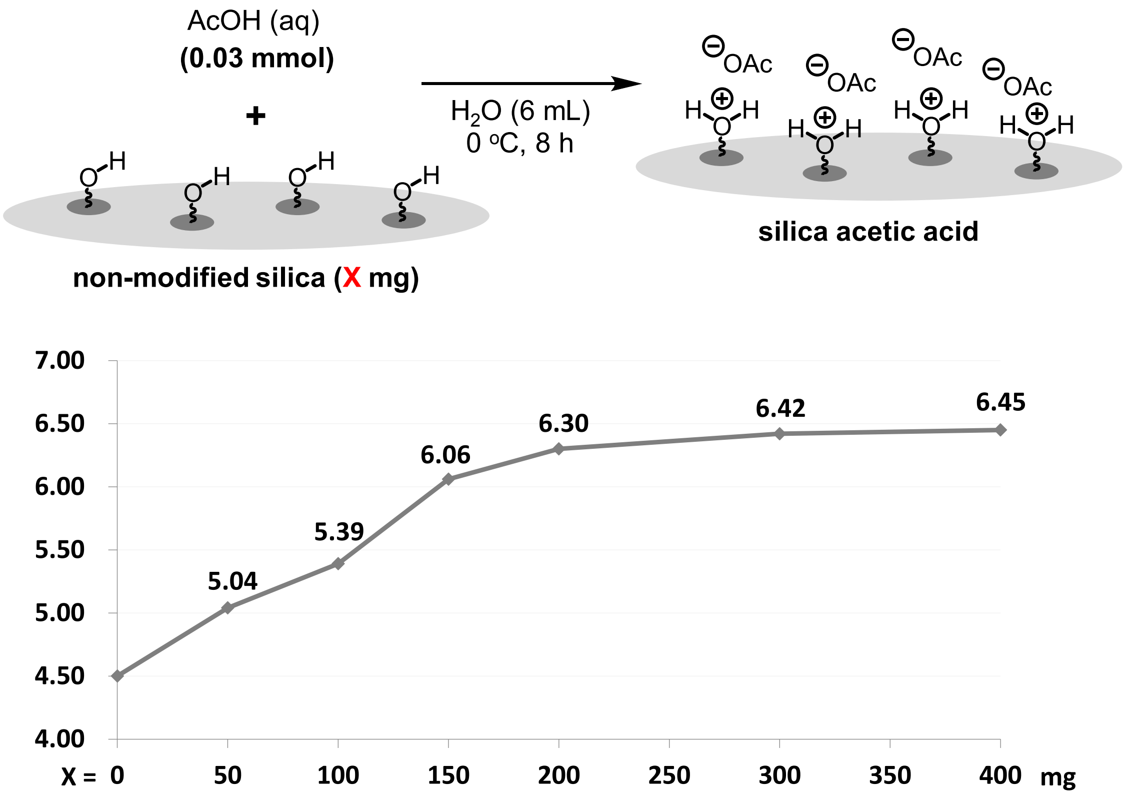

Figure S4. pH of silica acetic acid. As the amount of silica increases, the pH value also increased.

The zeta-potential of a silica slurry (200 mg, initial zeta-potential without adding AcOH: –34.8 mV) was measured with sequential addition of the indicated amount of acetic acid (**Y** equiv. based on **1**) in 6.0 mL of water. The results are shown in Figure S5. The addition of 1.0 equiv. acetic acid into the silica slurry resulted in an increase of zeta-potential up to –13.1 mV.

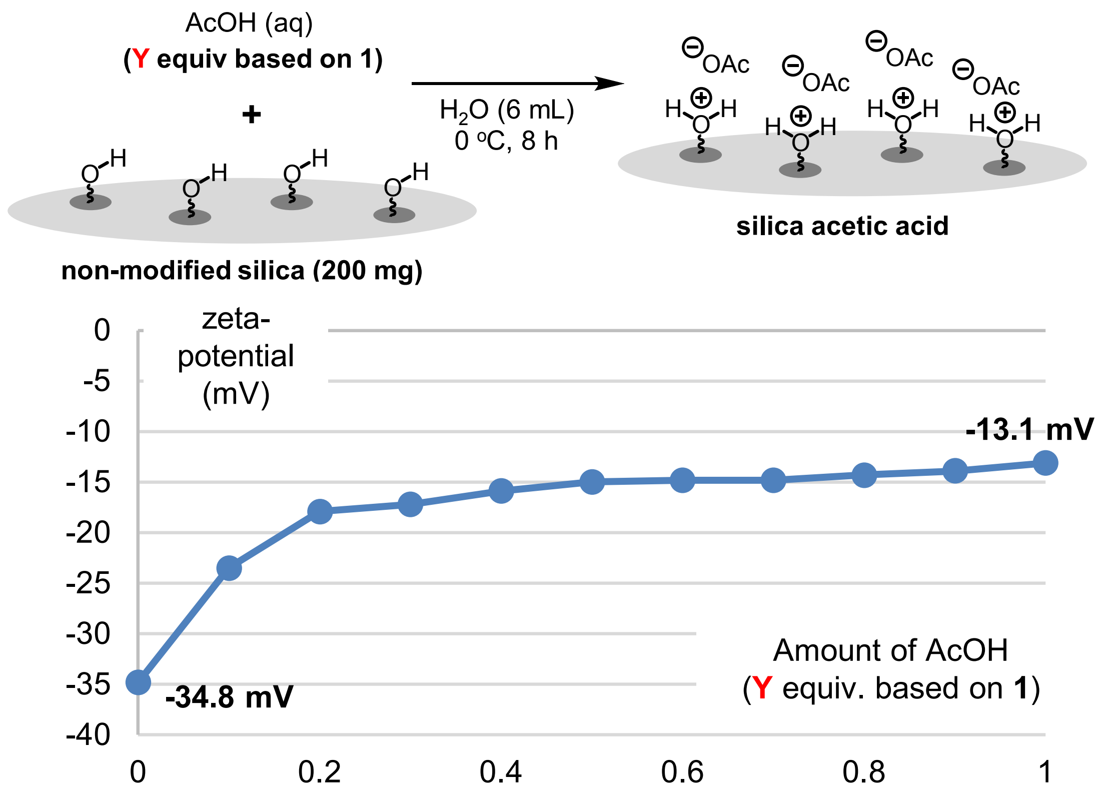

Figure S5. Zeta potential of silica slurry.

^{1}H and ^{13}C NMR spectra for new compounds

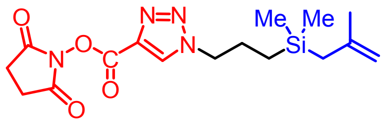

(1)

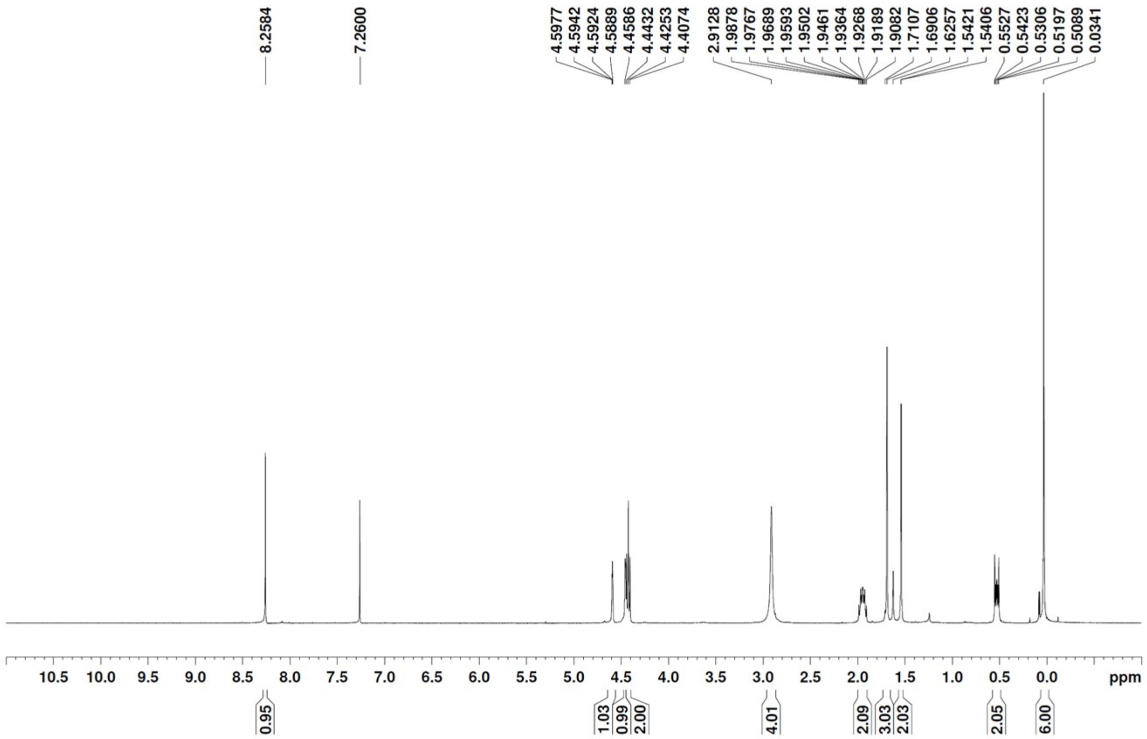

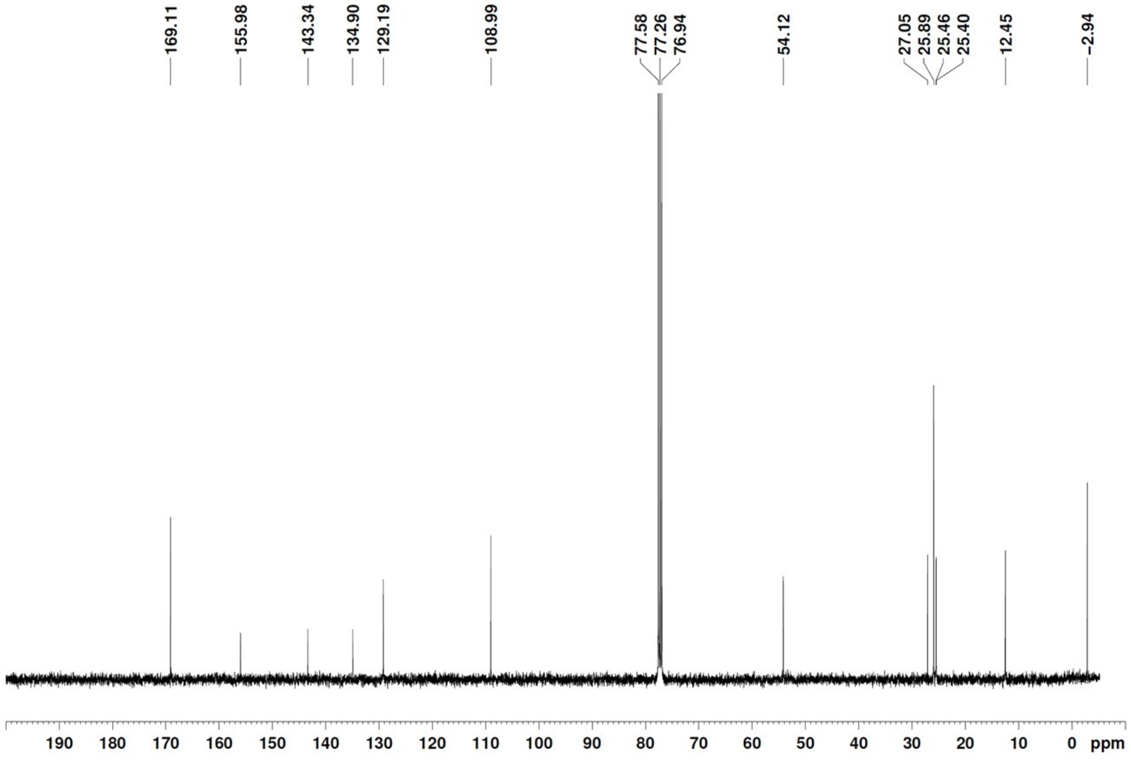

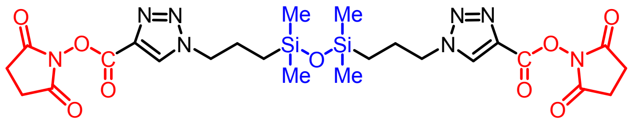

N=N
Me
Me
Si
O
Si
Me
Me
N=N

(2)

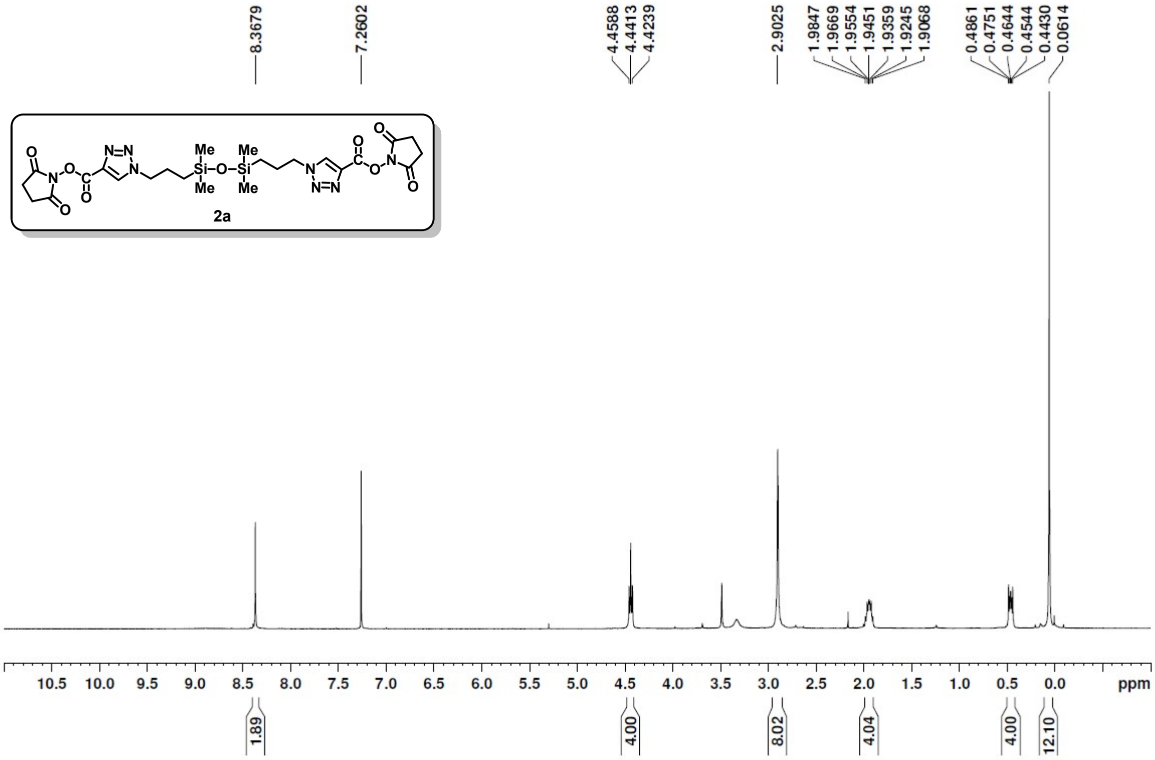

8.3679
7.2602
4.4588
4.4413
4.4239
2.9025
1.9847
1.9669
1.9554
1.9451
1.9359
1.9245
1.9068
0.4861
0.4751
0.4644
0.4544
0.4430
0.0614
2a
10.5 10.0 9.5 9.0 8.5 8.0 7.5 7.0 6.5 6.0 5.5 5.0 4.5 4.0 3.5 3.0 2.5 2.0 1.5 1.0 0.5 0.0 ppm
1.89
4.00
8.02
4.04
4.00
12.10

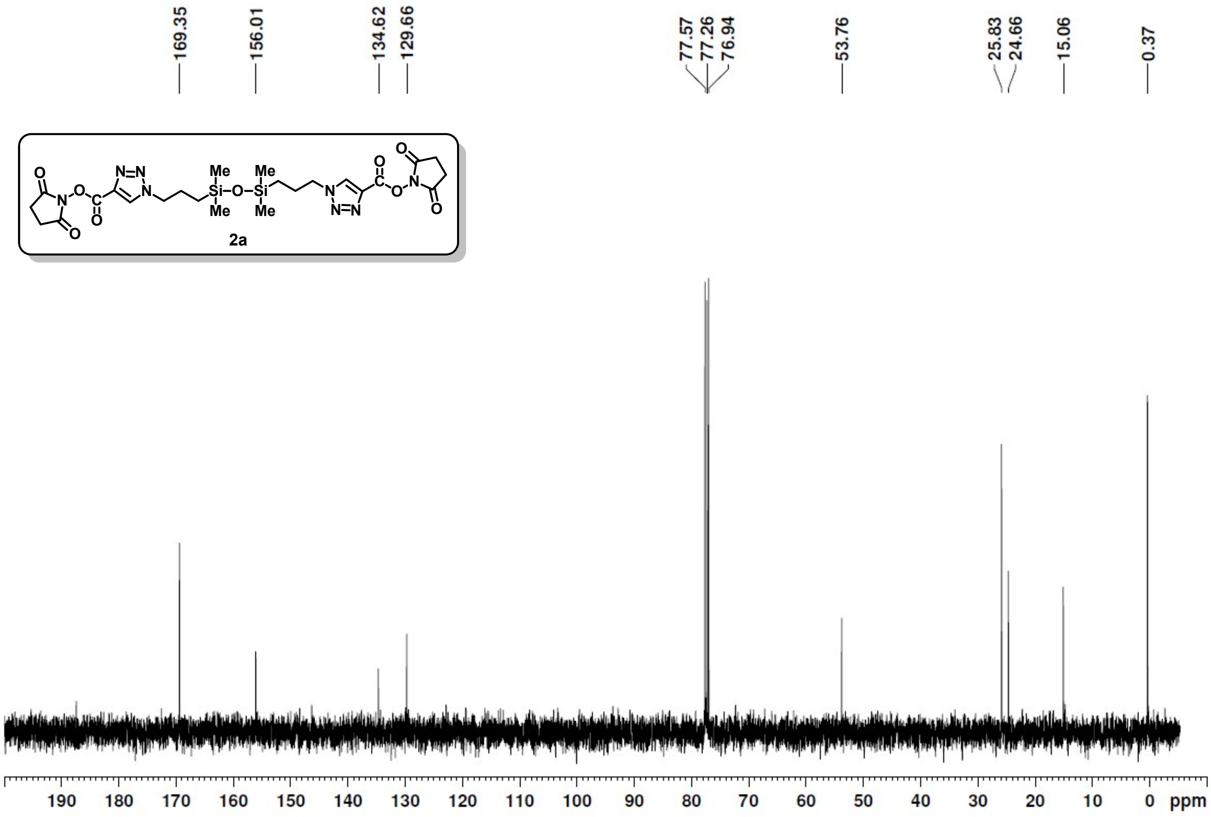

169.35
156.01
134.62
129.66
77.57
77.26
76.94
53.76
25.83
24.66
15.06
0.37
2a
190 180 170 160 150 140 130 120 110 100 90 80 70 60 50 40 30 20 10 0 ppm
